# Fusobacterium necrophorum Orbital Cellulitis With Intraconal Abscess

**DOI:** 10.7759/cureus.41415

**Published:** 2023-07-05

**Authors:** Benjamin P Otte, Justin P Harris, Alexandra J Schulte, Brett W Davies, Wesley L Brundridge

**Affiliations:** 1 Ophthalmology, San Antonio Uniformed Services Health Education Consortium, San Antonio, USA

**Keywords:** intraconal, allergic fungal sinusitis, orbital abscess, fusobacterium necrophorum, orbital cellulitis

## Abstract

A 19-year-old male presented to the emergency department with progressive right eye proptosis and was subsequently diagnosed with bacterial orbital cellulitis and acute on chronic allergic fungal sinusitis. He experienced brief symptomatic improvement after endoscopic sinus surgery, initiation of antibiotics, and steroid treatment; however, he re-presented five days after discharge with significantly worsened symptoms and no light perception in the right eye. Cultures resulted in Aspergillus and *Fusobacterium necrophorum*, a rare, aggressive etiology of bacterial orbital cellulitis. He developed an intraconal abscess requiring multiple orbitotomies for decompression and abscess drainage. To our knowledge, only eight prior cases of *F. necrophorum* orbital cellulitis have been reported in the literature (excluding the present case) and our patient is the first case of this organism causing an intraconal abscess. The authors discuss the importance of early recognition and close follow-up of *F. necrophorum *orbital infections.

## Introduction

*Fusobacterium necrophorum* is an obligate anerobic Gram-negative bacteria that can be found in the normal flora of the oropharynx. It is considered the primary organism responsible for post-anginal septicemia, or Lemierre’s syndrome. It is also a rare etiology of orbital cellulitis with only eight previously reported cases in the literature [1-7]. All reported cases of *F. necrophorum* orbital cellulitis were associated with sinus disease. Four of eight cases progressed to intracranial involvement and two developed epidural abscesses. Six of eight cases resulted in extraconal orbital abscess formation, and none reported intraconal abscess formation. The case below details the first reported case of *F. necrophorum* infection with abscess formation within the muscle cone resulting in severe, long-term vision loss. The collection and evaluation of protected health information were Health Insurance Portability and Accountability Act (HIPAA) compliant, and the patient provided written consent for participation in this case report to include the use of photographs and radiographs. This report was prepared to adhere to the ethical principles outlined in the Declaration of Helsinki.

## Case presentation

**Figure 1:**
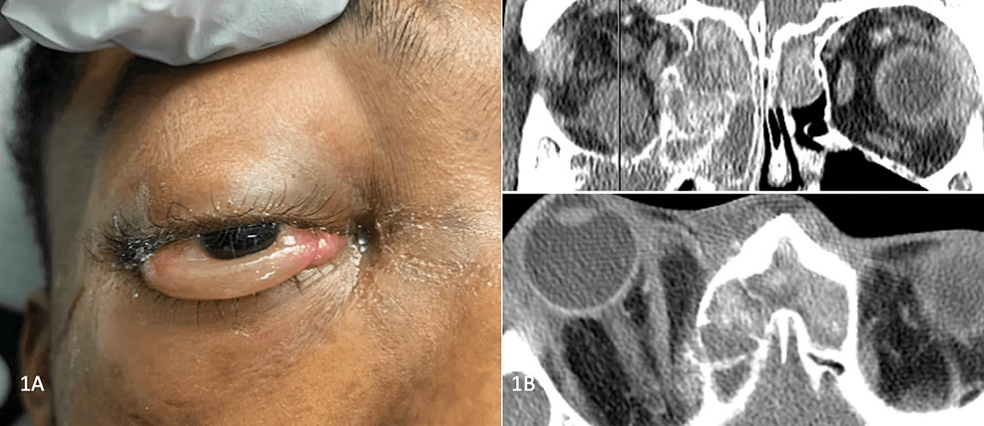
Initial presentation - 1A. Photograph of the right eye with chemosis and proptosis. 1B. Initial CT scan coronal and axial views.

A 19-year-old male presented to the emergency department with progressive right eye proptosis, pain with eye attempted eye movements, binocular diplopia in all gazes but worse in attempted upgaze, mucopurulent nasal discharge, and sinus congestion. He had a remote history of a right orbital floor fracture five years prior that did not require surgical intervention. The patient was otherwise healthy without other ocular histories. His visual acuity was 20/20 bilaterally, intraocular pressures (IOPs) were normal, and there was no relative afferent pupillary defect (APD). His exam was notable for right-sided proptosis, severe limitation of right eye movement in all directions, and inferior chemosis (see Figure 1A). Hertel exophthalmometry showed 10-mm proptosis in the right compared to the left. Initial CT imaging showed opacification of the bilateral frontal, right ethmoid, and right maxillary sinuses with areas of hyper-attenuation suggestive of fungal sinusitis, suspected polyp obstructing the middle meatus, and direct extension vs fungal invasion of the extraconal orbital space (see Figure 1B). Significant proptosis with straightening of the right optic nerve was also evident on CT imaging, although there was no apparent subperiosteal or other orbital abscess observed at this time. He underwent functional endoscopic sinus surgery (FESS) and right medial wall orbital decompression with otorhinolaryngology, during which tissue biopsies and cultures were obtained. Eosinophilic mucin and extensive nasal polyps consistent with fungal allergic sinusitis were observed intraoperatively and debrided. A tissue sample of the maxillary sinus contents was positive for Aspergillus niger; however, there was no evidence of invasive fungal disease or deep tissue involvement in other samples sent for pathology review. During hospitalization, he received broad-spectrum IV antibiotics including vancomycin, ceftriaxone, amphotericin B, as well as oral metronidazole to treat both allergic fungal sinusitis and suspected secondary bacterial orbital cellulitis. After five days his symptoms were improving, and his relative right-sided proptosis had decreased from 10 to 6 mm; he was discharged on oral antibiotics and steroids.

**Figure 2:**
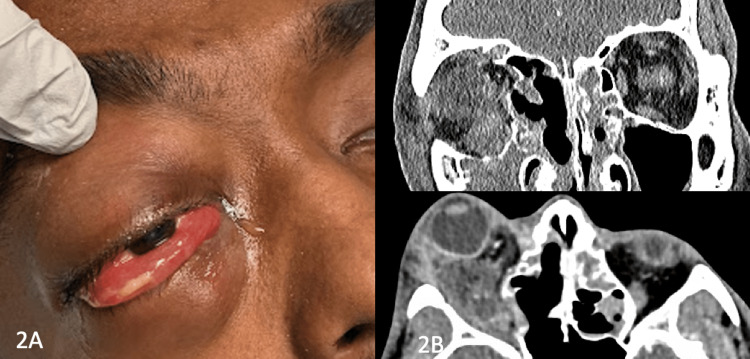
Second presentation - 2A. Photograph of the right eye. 2B. CT scan coronal and axial views demonstrating worsened orbital inflammation/infection.

The patient missed his follow-up appointment but reported his symptoms were reportedly improving via telephone. Five days post-discharge, he re-presented to the emergency department reporting an acute decrease in vision overnight with worsening right eye swelling and pain. His visual acuity in the right eye was no light perception (NLP), he had a right relative APD and his IOP was 43 mmHg OD. He had significant proptosis, chemosis, and limited movement of the right eye (see Figure 2A). An emergent lateral canthotomy and cantholysis were performed during which purulent fluid was expressed from the canthotomy wound. New cultures were obtained and the patient underwent repeat orbital CT imaging which demonstrated worsened cellulitis, an inferior post-septal abscess, and significant lateral phlegmon (see Figure 2B). Cultures from his prior hospitalization and repeat cultures resulted in both Aspergillus and *F. necrophorum*.

**Figure 3:**
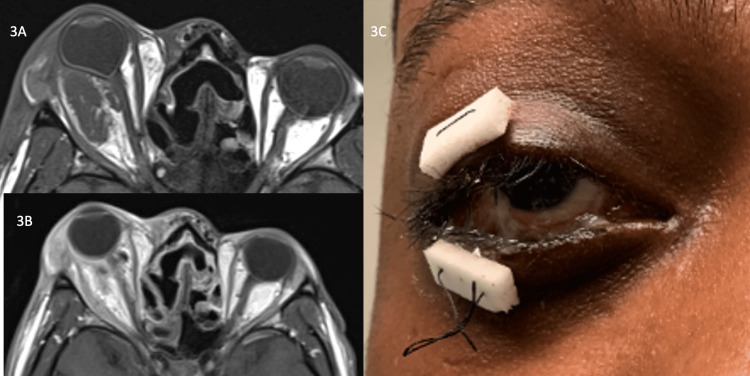
Hospital stay - 3A. MRI orbits axial view demonstrating intraconal abscess with tenting of the globe and straightening of the optic nerve; 3B. MRI following treatment; 3C. Photograph of the right eye following treatment showing improvement in proptosis and chemosis.

In consultation with the Infectious Disease service, the patient was started on the same broad-spectrum antibiotic regimen as his previous hospitalization (IV vancomycin, IV ceftriaxone, IV amphotericin B, and oral metronidazole). Culture sensitivities were obtained, and the Fusobacterium was found to be beta-lactamase negative. The patient underwent an initial orbitotomy via a transconjunctival approach to drain the abscess with intraconal dissection between the inferior and lateral rectus muscles, which also revealed purulence in the intraconal space. Necrotic tissue was debrided until healthy, bleeding tissue was observed. The wound was irrigated with an antibiotic solution and left open. Subsequent CT imaging the day after his first orbitotomy revealed an interval increase in phlegmon, as well as partial opacification of the bilateral maxillary sinuses and left ethmoid sinus. MRI imaging showed a 3.7 cm x 2.2 cm x 2.7 cm intraconal abscess with evidence of optic nerve ischemia and orbital apex involvement (see Figure 3A). A second orbitotomy occurred two days after the first orbitotomy during which more purulence was evacuated and a Penrose drain was left in place. Post-operative MRI showed an interval decrease in abscess size. The wound was repeatedly irrigated with polymyxin antibiotic solution via the Penrose drain while continuing the systemic antibiotic regimen. Hertel exophthalmometry was utilized to monitor the patient’s proptosis while hospitalized and four days following his second orbitotomy he was noted to have an acute increase in proptosis with a 9-mm difference and interval increase of abscess size on MRI. He was taken for a third orbitotomy with intraconal dissection between the inferior rectus and medial rectus muscles. More purulence was expressed, the wound was again irrigated, a drain was left in place, and a temporary tarsorrhaphy was placed to limit exposure given his continued chemosis. His proptosis decreased to a 4-mm difference by Hertel exophthalmometry following the third orbitotomy. Meropenem replaced ceftriaxone in his antibiotic regimen, oral prednisone was initiated, and serial MRIs showed gradual resolution of the intraconal abscess over several days (see Figure 3B). A peripherally inserted central catheter (PICC) line was placed and the patient was discharged on a three-week course of IV vancomycin and meropenem as well as oral metronidazole. His chemosis and proptosis continued to improve (see Figure 3C) with a 3-mm difference at discharge and a 2-mm difference one day after discharge. His right eye visual acuity remained NLP with residual right eye motility deficits six months post-discharge.

## Discussion

The most common causative pathogens of bacterial orbital cellulitis are Gram-positive Staphylococcus and Streptococcus, while orbital cellulitis secondary to the Gram-negative, obligate anerobe Fusobacterium is rare and can follow an aggressive clinical course [1, 8]. Sinusitis is a common etiology of orbital cellulitis with direct extension of pathogens from the upper respiratory tract through the paranasal sinuses into the orbit. Development of an abscess secondary to orbital cellulitis is thought to be an uncommon entity, although there is a wide variation in estimated incidences ranging from 7% to 83% [1, 9]. Orbital abscesses appear to occur at a relatively high rate when *F. necrophorum* is the offending pathogen, with abscesses developing in seven of the nine (78%) reported cases of *F. necrophorum* orbital cellulitis, including the present case, which is the only reported instance of associated intraconal abscess [1-7]. 

Studies of Fusobacteria bacteremia have found an annual incidence of 0.55/100,000, of which the species *F. necrophorum* accounted for only 25% of cases [10-11]. *F. necrophorum* bacteremia more commonly affects young, otherwise healthy adults, with a median age of 21 years [10]. The median age of the nine reported cases of *F. necrophorum* orbital cellulitis was 18 years (mean 16.3 years). Of these nine cases, visual acuity was 20/20 after treatment in four cases, 20/30 in two cases, and NLP in the current case; two cases report full recovery without noting final visual acuity [1-7]. The worst presenting visual acuity was Hand Motion vision in a patient case reported by Escardó et al., who eventually recovered to 20/30 vision following 30 days of IV antibiotic therapy [2]. The IV antibiotic courses ranged from six days to approximately six weeks followed by oral antibiotic courses. Patients received various broad-spectrum antibiotic regimens, with commonly used agents including a third-generation cephalosporin (e.g., ceftriaxone), vancomycin, meropenem, and metronidazole. Long-term IV antibiotic therapy resulted in largely favorable outcomes. In the current case, however, the patient initially received a five-day course of IV antibiotic therapy prior to the transition to oral antibiotics after which his clinical status deteriorated rapidly. Per the patient’s report, he experienced a sudden decrease in vision overnight, essentially waking up with NLP vision. The patient’s poor visual outcome was likely secondary to his development of a large intraconal abscess and associated elevated IOPs resulting in optic nerve damage. Intraconal abscess is a rare entity due to the barrier provided by the extraocular muscles and connective tissue surrounding the intraorbital fat. Surgery involving the periorbita, such as that that occurred with our patient who underwent FESS and medial orbital wall decompression, increases the risk of introducing pathogens into the intraconal space. 

At the initial visit, *F. necrophorum* had not yet been identified as the responsible pathogen, and his clinical picture was complicated by the presence of allergic fungal (Aspergillus) rhinosinusitis obstruction likely leading to the secondary Fusobacterium infection. Fusobacterium species are difficult to grow and often takes more than three to five days before growth is visible on culture media [12]. In the current case, Fusobacterium was observed on culture three days after re-presentation to the emergency department. Early surgery may be necessary to treat the patient’s sinus disease and/or evacuate abscesses. Patients underwent sinus surgery in six of the nine cases, and six of the seven patients with orbital abscesses underwent surgical evacuation. Our patient required three separate orbitotomies to evacuate the intraconal abscess and control the spreading infection which approached the cavernous sinus. The quick re-formation of the abscess following initial orbitotomy suggests the need for a more aggressive opening of the intraconal space to allow for better drainage and debridement. Early drain placement during the first orbitotomy to facilitate drainage and allow bedside irrigation would likely have been beneficial. 

## Conclusions

This case provides an example of the aggressive nature of this rare etiology of orbital cellulitis, which resulted in a rapid decline in vision and the development of an intraconal abscess despite broad-spectrum antibiotic therapy and sinus surgery. The rapid progression following the transition to oral antibiotics after the initial five-day IV antibiotic course suggests prolonged IV antibiotics may be necessary. Early MRI imaging and surgical debridement may also be necessary given this organism’s predilection for abscess formation.
